# Heme Oxygenase-1 Deletion Affects Stress Erythropoiesis

**DOI:** 10.1371/journal.pone.0020634

**Published:** 2011-05-31

**Authors:** Yu-An Cao, Sophie Kusy, Richard Luong, Ronald J. Wong, David K. Stevenson, Christopher H. Contag

**Affiliations:** 1 Department of Pediatrics, Stanford University School of Medicine, Stanford, California, United States of America; 2 Department of Comparative Medicine, Stanford University School of Medicine, Stanford, California, United States of America; University of Louisville, United States of America

## Abstract

**Background:**

Homeostatic erythropoiesis leads to the formation of mature red blood cells under non-stress conditions, and the production of new erythrocytes occurs as the need arises. In response to environmental stimuli, such as bone marrow transplantation, myelosuppression, or anemia, erythroid progenitors proliferate rapidly in a process referred to as stress erythropoiesis. We have previously demonstrated that heme oxygenase-1 (HO-1) deficiency leads to disrupted stress hematopoiesis. Here, we describe the specific effects of HO-1 deficiency on stress erythropoiesis.

**Methodology/Principal Findings:**

We used a transplant model to induce stress conditions. In irradiated recipients that received *hmox* ^+/−^ or *hmox* ^+/+^ bone marrow cells, we evaluated (i) the erythrocyte parameters in the peripheral blood; (ii) the staining intensity of CD71-, Ter119-, and CD49d-specific surface markers during erythroblast differentiation; (iii) the patterns of histological iron staining; and (iv) the number of Mac-1^+^-cells expressing TNF-α. In the spleens of mice that received *hmox* ^+/−^ cells, we show (i) decreases in the proerythroblast, basophilic, and polychromatophilic erythroblast populations; (ii) increases in the insoluble iron levels and decreases in the soluble iron levels; (iii) increased numbers of Mac-1^+^-cells expressing TNF-α; and (iv) decreased levels of CD49d expression in the basophilic and polychromatophilic erythroblast populations.

**Conclusions/Significance:**

As reflected by effects on secreted and cell surface proteins, HO-1 deletion likely affects stress erythropoiesis through the retention of erythroblasts in the erythroblastic islands of the spleen. Thus, HO-1 may serve as a therapeutic target for controlling erythropoiesis, and the dysregulation of HO-1 may be a predisposing condition for hematologic diseases.

## Introduction

Erythropoiesis, the development of mature red blood cells (RBCs), originates from pluripotent hematopoietic stem cells (HSCs) and progresses through erythroblasts to the formation of RBCs. Just prior to maturation, erythroblasts extrude their nucleus and mature into reticulocytes. As reticulocytes, they exit the bone marrow and enter blood capillaries to participate in oxygen transport. Homeostatic erythropoiesis maintains normal hematocrit levels and occurs as needed to replace old and damaged erythrocytes. In response to special needs, such as after bone marrow (BM) transplantation, myelosuppression, or anemia, stress erythropoiesis occurs as the body attempts to increase the production of erythrocytes. This process occurs in part within the spleen [1] and is regulated by several factors that are shared with homeostatic erythropoiesis. These include erythropoietin, iron, cytokines, cellular regulators, and adhesion molecules. Factors that distinguish homeostatic and stress hematopoiesis are less well described.

The heme oxygenase-1 enzyme (HO-1) is encoded by the *hmox-1* gene and is an inducible stress enzyme that catalyzes heme oxidation into carbon monoxide (CO), free ferrous iron, and biliverdin. The free iron from this reaction is sequestered by ferritin, and the biliverdin is rapidly converted into bilirubin by biliverdin reductase [2]. CO is a diffusible regulator that has been linked to the regulation of numerous cellular and tissue functions [3], [4]. HO-1 facilitates iron reutilization in mammals [5] and modulates the expression of cytokines [6] and adhesion molecules [7], [8]. HO activity has been implicated in the control of inflammation, immune regulation and organ transplantation [9], [10], [11]. Genetic knockout (KO) of HO-1 results in partial embryonic lethality and leads to a number of hematological disorders in surviving mice, including anemia, hypoferremia and tissue accumulation of iron [5], [10]. In a rare human case, HO-1 deficiency was associated with thrombocytosis, coagulation abnormalities, persistent hemolytic anemia, iron deposition in tissues, and premature death [12], [13].

We previously reported that HO-1 deficiency leads to disrupted stress hematopoiesis of stem cells and progenitors [14]. Mice lacking one allele of HO-1 (*hmox* ^+/−^) showed accelerated hematopoietic recovery from myelotoxic injury. However, *hmox* ^+/−^ HSCs were ineffective in the radioprotection and serial repopulation of myeloablated recipients. In this study we observed a threshold for effective transplantation of HO-1 knockout bone marrow; too few cells led to hematopoietic failure due to stem cell depletion, transplantation using cell numbers above this threshold were successful, but serial transplants from these recipient animals also resulted in hematopoietic failure.

Here we describe the effects of HO-1 deficiency specifically on stress erythropoiesis using cell numbers above the previously described threshold. We used a transplant model to induce stress conditions and demonstrated that HO-1 deletion affects stress erythropoiesis. In the spleen, HO-1 participates in proper erythroblast differentiation and optimal iron reutilization. Its deficiency increases the number of TNF-α-expressing cells and leads to a decrease of the CD49d levels in proerythroblasts. These findings suggest that HO-1 deficiency might cause a premature release of erythroblasts into the circulation.

## Results

### 1- HO-1 haploinsufficiency disrupts erythrocyte parameters in the peripheral blood

HO-1-deficient mice show a high level of embryonic lethality [5], and therefore the effects of HO-1 haploinsufficiency were studied as a means of assessing the intrinsic contribution of HO-1 in stress erythropoiesis following transplantation. Whole BM from either *hmox* ^+/+^ or *hmox* ^+/−^ mice was transplanted into lethally irradiated recipient mice.

**Figure 1:**
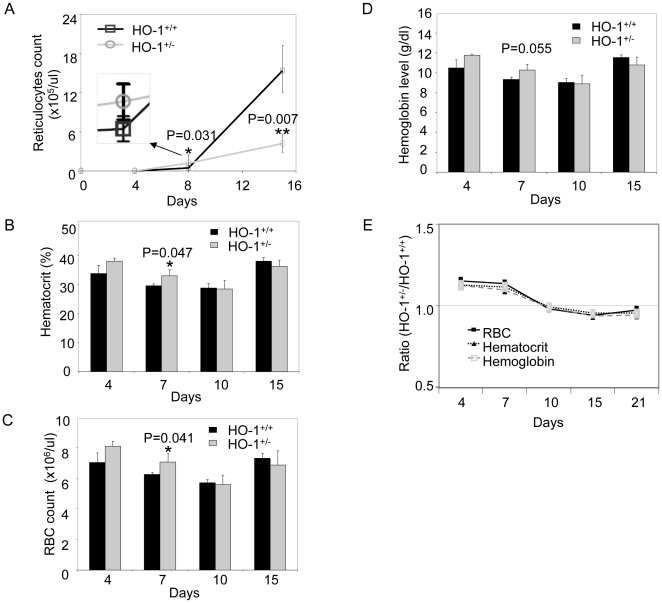
HO-1 haploinsufficiency disrupts erythrocyte parameters in the peripheral blood. Lethally irradiated mice were transplanted with 2×10^7^ *hmox* ^+/+^ or *hmox* ^+/−^ BM cells. Peripheral blood was drawn at each given time-point post-engraftment and was analyzed for the reticulocyte count (A), hematocrit percentage (B), RBC count (C), and hemoglobin level (D). The ratios of the RBC counts, hematocrit percentages, and hemoglobin levels of the *hmox* ^+/−^ to *hmox* ^+/+^ genotypes are plotted in (E) using the same data shown in (B-D). The mean ± SEM is shown for five mice per genotype; *P≤0.05, **P≤0.01.

Eight days after engraftment, the reticulocyte counts increased rapidly in recipients of *hmox* ^+/−^ BM cells, reaching a level of ∼250% of those in the recipients of *hmox* ^+/+^ BM cells (1.22±0.48×10^5^ per uL versus 0.47±0.14×10^5^ per uL; n = 5, *P = 0.031) (Figure 1A). In contrast, 15 days after engraftment, the reticulocyte counts in recipients of *hmox* ^+/−^ BM cells were only 27% of those observed in the mice that received *hmox* ^+/+^ BM cells (4.22±1.35×10^5^ per uL versus 15.38±3.75×10^5^ per uL; n = 5, **P = 0.007) (Figure 1A). This is consistent with our previous report in which we demonstrate accelerated hematopoiesis that was not sustainable after transplant [14].

We also observed elevated hematocrits in mice that received *hmox* ^+/−^ BM cells relative to those that received *hmox* ^+/+^ BM cells. At day 7, mice that received *hmox* ^+/−^ BM cells had statistically greater hematocrit levels than recipients of wild-type cells (33.0±2.2% versus 29.6±0.5%; *P = 0.047) (Figure 1B). However, at day 15, the hematocrits from recipients of either *hmox* ^+/−^ or *hmox* ^+/+^ BM cells (36.4±2.1% versus 38.2±1.2%) (Figure 1B) were lower than those in the non-transplanted mice (39.0% versus 47.0%, data not shown).

These data suggest that there was early-accelerated erythropoiesis in the recipients of *hmox* ^+/−^ BM cells. However, this level of erythropoiesis could not be sustained, and the number of immature erythrocytes declined prior to a full recovery from the blood loss.

Mice that received *hmox* ^+/−^ BM cells had statistically more RBCs (7.1±0.5×10^6^ per uL versus 6.2±0.1×10^6^ per uL; n = 5, *P = 0.041) at day 7 (Figure 1C), whereas their hemoglobin levels were slightly, but not statistically significantly, higher (10.2±0.6 g/dL versus 9.3±0.2 g/dL; n = 5, P = 0.055) compared to those of mice that received *hmox* ^+/+^ BM cells (Figure 1D).

The ratios of the RBC count, hematocrit, and hemoglobin levels between the mice that received *hmox* ^+/−^ and *hmox* ^+/+^ BM cells decreased gradually over time from 1.13–1.15 (at day 4) to 0.92 - 0.95 (at day 15) (Figure 1E). These data show that there are more *hmox* ^+/−^ BM cell-derived erythrocytes in the peripheral blood than *hmox* ^+/+^ BM cells from day 4 to 10, and the opposite from day 10 to 21. This suggests that *hmox* ^+/−^ BM cell-derived erythrocytes might appear earlier in the peripheral blood than those from *hmox* ^+/+^ BM cells.

### 2- HO-1 participates in proper erythroblast differentiation

**Figure 2:**
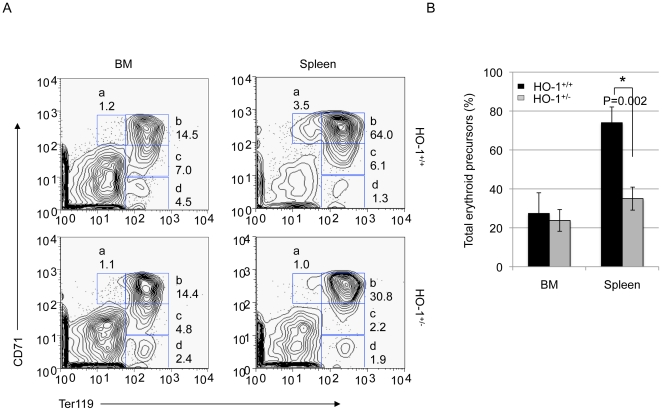
HO-1 participates in proper erythroblast differentiation. (A) Representative FACS profiles of freshly isolated BM cells (left panels) and splenic cells (right panels) from *hmox* ^+/+^ or *hmox* ^+/−^ BM cell recipients at day 15 post-transplantation are shown. Cells were labeled with PE-conjugated anti-CD71 and PE-Cy7-conjugated anti-Ter119. Dead cells (stained positive with propidium iodide) and enucleated erythrocytes (with low forward scatter) were excluded from the analysis. The regions (from left to right and then from top to bottom) distinctly differentiate four classes of erythroid precursors (from the earlier to the most mature): proerythroblasts (Ter119^med^CD71^high^) in region a, basophilic erythroblasts (Ter119^high^CD71^high^) in region b, late basophilic and polychromatophilic erythroblasts (Ter119^high^CD71^med^) in region c, and orthochromatophilic erythroblasts (Ter119^high^CD71^low^) in region d. The engraftment of HO-1-deficient BM cells modifies the FACS profile of the erythroblastic BM and splenic cells. (B) The frequencies of total erythroid precursors found in regions a, b, c and d in the BM and the spleens of *hmox* ^+/+^ or *hmox* ^+/−^ BM cell recipients are shown. The transplantation of HO-1-deficient BM cells leads to a decrease in the erythroid precursor population. The mean ± SEM is shown for six mice per genotype; *P≤0.05.

The spleen and BM serve as reserves for accelerated hematopoiesis under conditions of hematopoietic stress. Loss of HO-1 did not change the total spleen and BM cellularities (data not shown). To further investigate whether HO-1 deficiency specifically affects stress erythropoiesis, we adopted a flow cytometric assay and analyzed cells from the BM and spleen of transplanted mice. Four classes of erythroid precursors can be identified by the staining intensities (low, medium (med), or high) of specific surface markers upon maturation, as shown in Figure 2 and as previously described [15]. Listed in order of the earliest to the most mature, these precursors are the early proerythroblast (Ter119^med^CD71^high^) identified in region a, the basophilic erythroblast (Ter119^high^CD71^high^) in region b, the late basophilic and polychromatophilic erythroblast (Ter119^high^CD71^med^) in region c, and the orthochromatophilic erythroblast (Ter119^high^CD71^low^) in region d.

At day 15 post-transplantation, the BM of mice engrafted with *hmox* ^+/−^ cells presented decreased numbers of polychromatophilic (regions c, ∼1.5-fold) and orthochromatophilic (region d, ∼1.9-fold) populations compared with control mice engrafted with *hmox* ^+/+^ cells (Figure 2A, left panels). Interestingly, the spleens of mice engrafted with *hmox* ^+/−^ cells presented decreased numbers of proerythroblast (region a, ∼3.5-fold), basophilic (region b, ∼2-fold) and polychromatophilic (region c, ∼2.7-fold) populations compared with the control animals (Figure 2A, right panel). Thus, the proportions of total erythroid precursors analyzed in the BM of *hmox* ^+/+^ and *hmox* ^+/−^ recipients were 27.4±10.6% and 23.8±5.6%, respectively (n = 6, P = 0.07) (Figure 2B). The proportions of total erythroid precursors analyzed in the spleens of *hmox* ^+/+^ and *hmox* ^+/−^ recipients were 74.0±8.1% and 35.0±5.9%, respectively (n = 6, *P = 0.002) (Figure 2B). Similar results were obtained at eight days post-transplantation (data not shown).

These data indicated that HO-1 deletion resulted in deficient erythroblast differentiation following transplantation of *hmox* ^+/−^ BM cells, both in the spleen and to a lesser extent in the BM. Given these data, we focused on the spleen as a site of stress erythropoiesis.

### 3- HO-1 participates in optimal iron re-utilization

Iron is an essential component of heme synthesis and hemoglobin formation. The majority of iron in erythropoiesis is reutilized in the breakdown of heme from senescent erythrocytes. Because HO-1 has an important iron-recycling role [5], we investigated whether iron reutilization is optimal in the spleen after the engraftment of HO-1-deficient BM cells.

**Figure 3:**
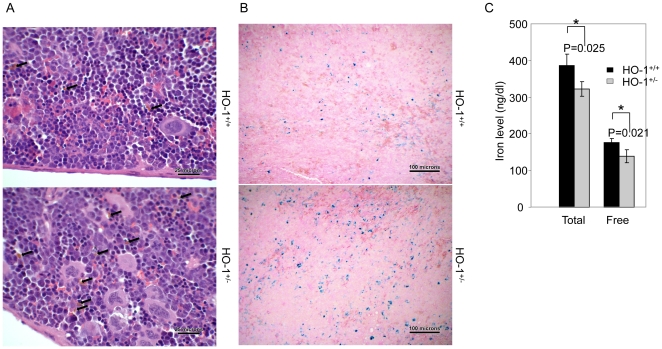
HO-1 participates in optimal iron re-utilization. (A) H&E staining of the spleen sections from *hmox* ^+/+^ or *hmox* ^+/−^ cell recipients at day 14 post-transplantation is shown. The arrows indicate hemosiderin-laden macrophages. The transplantation of HO-1-deficient BM cells leads to an increase of hemosiderin. The magnification is 400×. The photograph shown is a representative of three experiments. (B) Prussian blue iron staining of the spleen sections from *hmox* ^+/+^ or *hmox* ^+/−^ cell recipients at day 14 post-transplantation is shown. The transplantation of HO-1-deficient BM cells leads to an increase of Prussian blue-positive cells. The magnification is 100×. The photograph shown is a representative experiment out of the three that were performed. (C) The total and free iron levels in the splenic homogenates from *hmox* ^+/+^ or *hmox* ^+/−^ cell recipients at day 14 post-transplantation are shown. The transplantation of HO-1-deficient BM cells leads to a decrease of soluble iron. The mean ± SEM is shown for six mice per genotype; *P≤0.05.

To test this possibility, histological analysis of the spleen was performed 14 days post-transplantation. Hemosiderin-laden macrophages, accounted for approximately 2% of the total red pulp nucleated cell population in the spleens of mice that received *hmox* ^+/−^ BM cells, compared to ∼1% in mice that received *hmox* ^+/+^ BM cells (Figure 3A, upper levels; see Methods for quantification). Prussian blue staining showed an average of 2.6% of the splenic cross-sectional area to be positive for iron in the recipients of *hmox* ^+/−^ BM cells, compared to 0.9% in the recipients of *hmox* ^+/+^ BM cells (Figure 3B, lower levels).

The analysis of soluble iron in spleen homogenates showed the total and free iron levels of mice that received *hmox* ^+/−^ BM cells to be 323±21 and 144±18 ng/dL, respectively. This is 20% less compared to the 399±32 and 183±12 ng/g observed in the recipients of *hmox* ^+/+^ BM cells (n = 6, *P = 0.025 and *P = 0.021, respectively) (Figure 3C). No significant differences were observed in the plasma (data not shown).

Here, both the increase of the insoluble iron level (Figures 3A and 3B) and the decrease of the soluble iron level (Figure 3C) in the spleens of mice following HO-1^+/−^ BM cell transplantation suggest that HO-1 participates in optimal iron reutilization in spleens after transplantation.

### 4- HO-1 deficiency increases the number of TNF-α-expressing cells in the spleen

The proliferation and differentiation of erythroblasts occur in specialized niches known as erythroblastic islands, which are composed of central macrophages and erythroblasts at all stages of differentiation [16]. Central macrophages secrete cytokines such as TNF-α, which exerts an inhibitory effect on erythropoiesis [17], [18], [19], and HO-1 has been shown to suppress the effects of TNF-α [20], [21].

**Figure 4:**
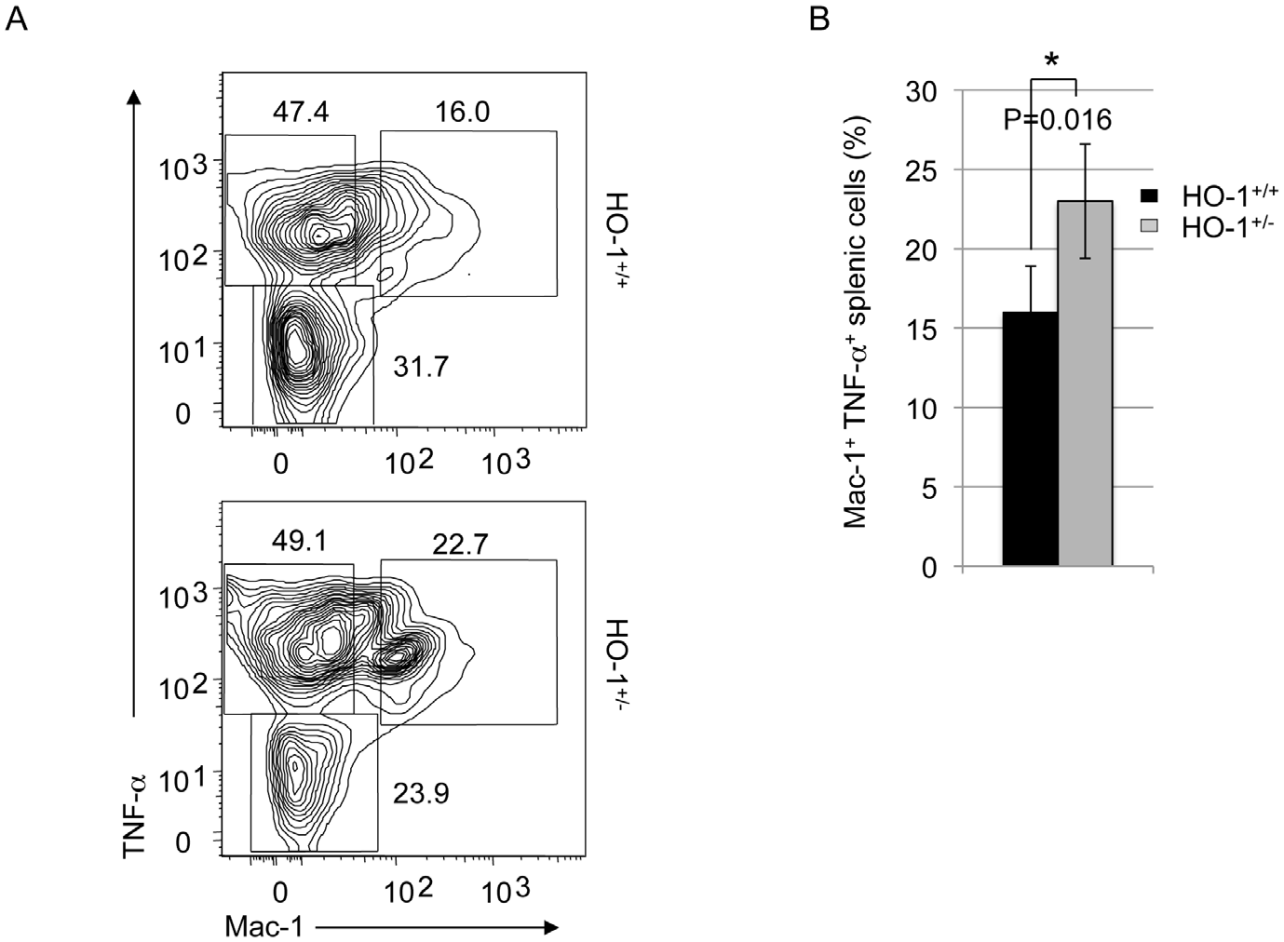
HO-1 deficiency increases the number of TNF-α-expressing cells. (A) Representative FACS profiles of freshly isolated splenic cells from *hmox* ^+/+^ or *hmox* ^+/−^ BM cell recipients at day 15 post-transplantation are shown. Cells were labeled with and PE-conjugated anti-TNF-α and PE-Cy7-conjugated anti-Mac-1. Dead cells (stained positive with propidium iodide) were excluded from the analysis. The engraftment of HO-1-deficient BM cells modifies the FACS profile of Mac-1 TNF-α-stained splenic cells. (B) The frequencies of Mac-1^+^ TNF-α^+^ splenic cells from *hmox* ^+/+^ and *hmox* ^+/−^ BM cell recipients are shown. The transplantation of HO-1-deficient BM cells leads to an increase of the Mac-1^+^ TNF-α^+^ splenic population. The mean ± SEM is shown for six mice per genotype; *P≤0.05.

To test whether HO-1 deficiency alters the expression of TNF-α in stress erythropoiesis, we assessed the number of Mac-1^+^-cells expressing TNF-α in the spleens of recipients following *hmox* ^+/−^ and *hmox* ^+/+^ BM cells transplantation (Figure 4A). At day 15 post-engraftment, 16.0±2.9% of Mac-1^+^ TNF-α^+^ cells were analyzed in the spleens of *hmox* ^+/+^ BM cells-recipients, compared to 22.7±3.6% in those of *hmox* ^+/−^ BM cells-recipients (n = 6; *P = 0.016) (Figure 4B). This 40% increase due to HO-1 deficiency suggests that TNF-α expression might be involved in the disrupted stress erythropoiesis in HO-1 deficiency.

### 5- HO-1 deficiency leads to a decrease of the CD49d level in splenic proerythroblasts

The adherence of erythroblasts to central macrophages within erythroblastic islands enables proper erythroblastic differentiation [22]. This cell-to-cell interaction is mediated by α4β1 integrin and its α-subunit CD49d in erythroblasts and vascular adhesion molecule-1 (VCAM-1) in macrophages [23].

Using a flow cytometric assay, we confirmed that CD49d expression decreased in a pattern following erythroblast maturation, from the highest expression in immature (Ter119^low^CD71^high^) cells to the lowest in the most differentiated erythroblasts (Ter119^high^CD71^low^) (Figure S1), consistent with previously reports [24].

**Figure 5:**
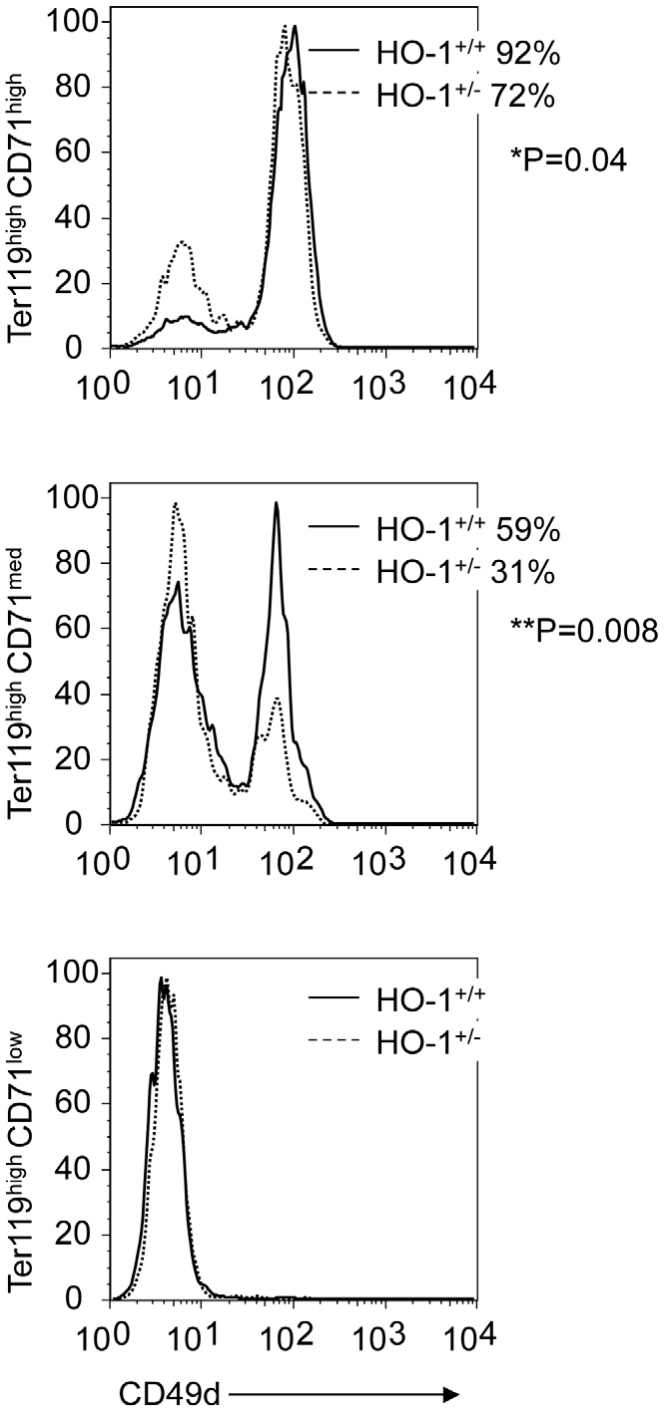
HO-1 deficiency leads to a decrease of the CD49d level in splenic proerythroblasts. Representative FACS histograms of basophilic (Ter119^high^CD71^high^), polychromatophilic (Ter119^high^CD71^med^), and orthochromatophilic (Ter119^high^CD71^low^) splenic erythroblasts from *hmox* ^+/+^ or *hmox* ^+/−^ BM cell recipients labeled with a FITC-conjugated anti-CD49d are shown at day 15 post-transplantation. The average frequencies of CD49d-positive cells in the different fractions of splenocytes are indicated for both HO-1 genotypes. The mean ± SEM is shown for six mice per genotype; *P≤0.05, **P≤0.01.

We then investigated the effect of HO-1 deficiency on the CD49d expression in splenic erythroblasts following BM transplantation. At day 15 post-transplantation, only 72% of *hmox* ^+/−^ Ter119^high^ CD71^high^ cells were CD49d^+^, compared to 92% of *hmox* ^+/+^ cells (n = 6, *P = 0.04) (Figure 5, upper panel). A more drastic deviation was shown in the Ter119^high^ CD71^med^ population, in which only 31% of *hmox* ^+/−^ cells were CD49d^+^ compared to 59% of *hmox* ^+/+^ cells (n = 6, **P = 0.008) (Figure 5, middle panel). All the cells were CD49d negative in the splenic Ter119^high^CD71^low^ populations (Figure 5, lower panel).

These results show that in this transplant model, HO-1 deficiency affects CD49d expression during splenic erythroblast maturation.

## Discussion

We have previously demonstrated that HO-1-deficient BM cells provide inadequate radioprotection of lethally irradiated mice [14], and we have suggested that the loss of one allele of HO-1 may be sufficient to maintain the steady-state metabolism of heme but insufficient under conditions of stress [14].

In this study, we documented for the first time the importance of HO-1 in stress erythropoiesis and address the mechanism of hematopoietic failure. Using a transplant model to induce stress conditions, we showed that HO-1 haploinsufficiency led to disrupted erythroblast differentiation. This resulted in the reduction, or loss, of immature red blood cells during differentiation from proerythroblasts to the orthochromatophilic erythroblast stage. Stress erythropoiesis is usually characterized by an increase in the absolute number of total Ter119^+^ erythroblasts [1]. Here, we showed that HO-1 deficiency led to a ∼50% decrease in the Ter119^high^ erythroblastic population. It is interesting to note that the disrupted erythroblast differentiation following the transplantation of *hmox* ^+/−^ BM cells was more dramatic in the spleen than in the BM. This spleen-selective change is consistent with the convention that stress erythropoiesis relies on a specialized population of stress erythroid progenitors that primarily reside in the spleen [25], [26], [27], [28], [29].

We also demonstrated a 20% decrease of the free iron levels and a 40% increase of the TNF-α+ cell count in the spleens of mice that received *hmox* ^+/−^ BM cells compared to those that received *hmox* ^+/+^ BM cells. These changes were not observed in the BM (Cao, unpublished data). It is still unclear whether the increased TNF-α expression is a feature of decreased stress erythropoiesis or if it is associated with increased numbers of macrophages due to decreased iron utilization. It has been recently shown that only a subset of macrophages express HO-1 [30]. The activation of macrophages by exposure to lipopolysaccaride (LPS) and either GM-CSF or M-CSF leads to the generation of TNF-α and IL-12p40-producing pro-inflammatory macrophages [M1 (GM-CSF)] or IL-10-producing anti-inflammatory macrophages [M2 (M-CSF)], respectively. A different iron metabolism gene signature is detected in the two macrophage types, with HO-1 being preferentially expressed by M2 (M-CSF) macrophages [30]. Thus, in our study, it appears that only a subset of macrophages might have been affected by the HO-1 haploinsufficiency.

We demonstrated that HO-1 deficiency leads to a decrease of the CD49d level in proerythroblasts. This finding suggests that HO-1 deficiency might cause a premature release of erythroblasts into the blood circulation. We speculate that the decreased levels of CD49d effect immature erythroblasts to result in disrupted erythroblast differentiation and an early release of these cells from erythroblastic islands, subsequently leading to inadequate stress erythropoiesis. Erythroblasts are known to express adhesion molecules that undergo dynamic variation during differentiation. These proteins mediate both erythroblast-to-erythroblast and erythroblast-to-macrophage interactions, as well as attachments to the extracellular matrix components. Two major receptors/counter-receptors identified as mediating the cell-to-cell interactions within erythroid islands are α4β1 integrin (VLA-4) in erythroblasts and VCAM-1 in central macrophages [23]. The α4β1 integrin has a critical role in erythropoiesis by holding maturing precursors in close association with the marrow stroma [31], [32], [33], [34], [35]. The α4 integrin sub-unit (CD49d) is essential to maintaining normal hematopoiesis in the spleen and BM microenvironments [36] and mediates the association between primitive erythroblasts and fetal liver-derived macrophages [37]. HO-1 has already been shown to modulate the expression of adhesion molecules [7], [8], and it is interesting to note that patients with sickle cell anemia have an increased level of CD49d expression [38], suggesting that a reduction in the adhesive properties may contribute to the premature release of erythrocytes into the peripheral blood.

We suggest here that decreased CD49d expression affects splenic HO-1-deficient erythroblasts to lead to their premature release into the circulation. Thus, the disrupted stress erythropoiesis described here might explain the ineffectiveness of *hmox* ^+/−^ BM cells in protecting lethally irradiated mice [14]. We propose that the HO-1 deficiency upset the self-renewal/differentiation balance toward improper differentiation, prematurely depleting the HSC reserve and releasing immature erythroblasts.

These results have implications in human hematological diseases and their treatment. The promoter of the human *hmox* gene is characterized by (GT)n repeats, with commonly occurring length polymorphisms affecting the gene expression that have been associated with a wide variety of diseases [39]. For example, shorter repeats are associated with increased susceptibility to some cancers and cerebral malaria, but these also have better liver and kidney transplant survival, whereas long GT repeat polymorphisms cause a lower expression of HO-1 but are associated with emphysema, miscarriages, and stroke [39]. It has been documented that patients with myelodysplastic syndrome (MDS) present macrophages with increased levels of TNF-α [40] and hematopoietic progenitors with reduced expression levels of CD49d [41]. It is intriguing that HO-1 deficiency shares some of the features of MDS, and it will be important to investigate whether the polymorphisms of HO-1 affect physiologic and pathologic human erythropoiesis in similar manners as in MDS patients. We anticipate that this research will not only lead to a better understanding of the role of HO-1 in stress hematopoiesis but will also suggest the clinical relevance of HO-1 as a target of novel therapies for the treatment of human hematological pathologies.

## Materials and Methods

### 1- Mice

FVB/NJ recipient mice were obtained from Charles River Laboratories (Wilmington, MA, USA). *hmox* ^−/−^ and *hmox* ^+/+^ mice were previously described [10]. All the experiments were set up when the animals were 10 weeks old. Comparisons of *hmox* ^+/−^ and *hmox* ^+/+^ mice were always conducted using littermates. The mice were housed in the Research Animal Facility at Stanford University, and all experiments were conducted under strict adherence to institutional guidelines, as approved by the Animal Care and Use Committee at Stanford University (APLAC #12323).

### 2- Purification of hematopoietic cells

Whole blood (200 µL) was drawn from the retro-orbital sinus into EDTA-coated microhematocrit tubes (Sarstedt, Numbrecht, Germany) and was sent to the diagnostic laboratory in the Department of Comparative Medicine at Stanford University for measurements of erythrocyte parameters. Blood counts and erythrocyte indices were determined using an automated hematology analyzer (Abbott Cell Dyne 3500, Abbott Park, IL, USA). To obtain splenic cells, the spleen was dissected, and a single cell suspension was generated by gently squishing the spleen in a small volume of phosphate buffered saline (PBS). To recover cells from the mouse BM, the marrow was flushed out of the tibia, femur, and humerus using a syringe with PBS, filtered through a 70-µm mesh filter (BD Biosciences, San Jose, CA, USA) to remove debris and pelleted by centrifugation. RBC contamination was removed using an RBC-lysis buffer containing ammonium chloride in 0.01 M Tris.

### 3- Bone marrow transplantation

In preparation for transplant, recipient mice (FVB/NJ) at two months of age were lethally irradiated (one single dose of 11 Gy). The following day, isolated BM cells (2×10^7^) from *hmox* ^+/−^ or *hmox* ^+/+^ mice were intravenously injected into the tail veins of recipient animals under methoxyflurane anesthesia. Under these conditions, over 98% of the cells in the BM and spleens of recipients are donor-derived at day 6 or later after transfer [14]. Thus, flow cytometric assays were performed with minimal interference from residual host cells, resolving the distinct stages of erythroid differentiation as previously described [15].

### 4- Phenotyping and flow cytometry

For erythroid cell subset analyses, splenic and BM cells were stained with PE-conjugated anti-CD71 (BD Biosciences), PE-Cy7-conjugated anti-Ter119 (eBioscience, San Diego, CA, USA), and FITC-conjugated anti-CD49d (Affinity BioReagents, Rockford, IL, USA). For TNF-α detection, splenic cells were first stained with PE-Cy7-conjugated anti-Mac-1 (BD Biosciences). After fixation and permeabilization, splenic cells were stained with PE-conjugated anti-TNF-α (CALTAG Laboratories, Burlingame, CA, USA). All stained cells were analyzed using the LSR instrument (BD Biosciences) and FlowJo software (Tree Star Inc., Ashland, OR, USA).

### 5- Histological staining for iron

Spleens were fixed in 4% paraformaldehyde, dehydrated in ethanol, embedded in paraffin, sectioned and mounted onto glass slides. Sections were stained either by hematoxylin and eosin (H&E) or Prussian blue according to standard protocols. High-resolution photomicrographs of six non-overlapping areas were taken at 200× magnification (2,590×1,920 pixels representing 0.37508 mm^2^) with a digital microscope camera system (Powershot G9, Canon, Lake Success, NY, USA; AxioVision 4.7.0.0, München, Germany). Each photomicrograph was imported into and processed by digital imaging software (UTHSCSA ImageTool for Windows 3.00, San Antonio, TX, USA). The total area of iron staining divided by the total area of the photomicrograph field (expressed as a percentage per 200× photomicrograph field) was determined for each of the six photomicrographs. The mean percentage of iron staining was determined by averaging the results from the six photomicrographs.

### 6- Iron level measurements

Spleens, which primarily contains erythroid cells, were homogenized in 19 volumes of PBS. Homogenates were centrifuged at 16,100×g for 2 min to remove cellular debris (including insoluble hemosiderin iron), and supernatants were collected to measure total and free iron. Iron measurements were performed and analyzed at Analytics Inc. (Gaithersburg, MD, USA).

### 7- Statistical analyses

Quantitative data are expressed as the mean ± SEM. All statistical analyses were performed on raw data for each group by one-way analysis of variance followed by a Student's t test. Differences among groups were considered to be significant if the probability of error was less than 0.05. The numbers of animals used for each analysis are mentioned in the figure legends, in addition to the effective P-values.

## Supporting Information

Figure S1 Representative FACS histogram of CD49d staining in splenic proerythroblasts (Ter119^med^CD71^high^), basophilic erythroblasts (Ter119^high^CD71^high^), polychromatophilic erythroblasts (Ter119^high^CD71^med^) and orthochromatophilic erythroblasts (Ter119^high^CD71^low^). The frequency of CD49d-positive cells decreases with erythroblast maturation.
(TIF)
