# Supplementary figures and images for: Heme Oxygenase-1 Deletion Affects Stress Erythropoiesis
Source: PLoS One. 2011 May 31;6(5):e20634. doi: 10.1371/journal.pone.0020634 (PMC3105104; doi:10.1371/journal.pone.0020634)

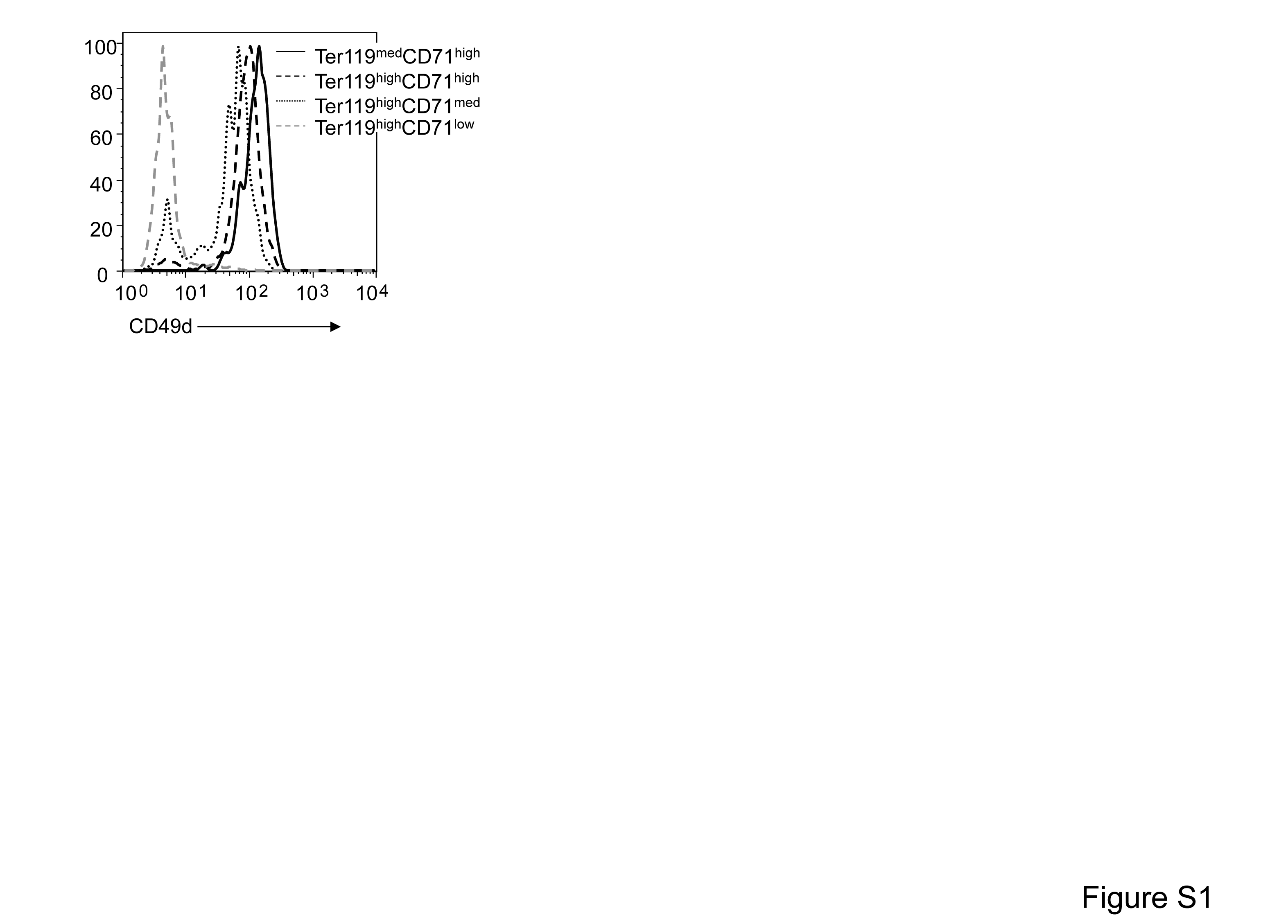

Supplement: Figure S1 — Representative FACS histogram of CD49d staining in splenic proerythroblasts (Ter119medCD71high), basophilic erythroblasts (Ter119highCD71high), polychromatophilic erythroblasts (Ter119highCD71med) and orthochromatophilic erythroblasts (Ter119highCD71low). The frequency of CD49d-positive cells decreases with erythroblast maturation. (TIF) [file pone.0020634.s001.tif]
